# Validated High Performance Thin Layer Chromatographic Determination and Content Uniformity Test for Rosiglitazone in Tablets

**DOI:** 10.4103/0250-474X.65034

**Published:** 2010

**Authors:** S. G. Walode, H. K. Chaudhari, M. S. Saraswat, A. V. Kasture, S. G. Wadodkar

**Affiliations:** Sinhgad Institute of Pharmaceutical Sciences, Kusgaon (BK), Lonavala, Pune-410 401, India; 1 Department of Pharmaceutical Sciences, RTM Nagpur University Campus, Amrawati Road, Nagpur-440 033, India

**Keywords:** HPTLC, caffeine, rosiglitazone, linearity, ruggedness

## Abstract

A simple, rapid, precise and economical high performance thin layer chromatographic method has been developed and validated for determination of rosiglitazone in its tablet dosage form using caffeine as an internal standard. It was performed on silica gel 60 GF_254_ thin layer chromatographic plates as a stationary phase using mobile phase methanol:toluene:chloroform:triethylamine (1:8:0.5:0.5 v/v/v/v) and the detection was carried out in the absorbance mode at 264 nm showing R_f_ value 0.31 for rosiglitazone and 0.52 for caffeine. The linear regression data curve shows good linear relationship in the concentration range 1.0-7.0 µg/µl. The content uniformity test was carried out as per USP specification of the content uniformity test of 85-115%. The percent drug estimated of rosiglitazone from two different marketed formulations were found to be in the range 99.83-100.21. The recovery of drugs was carried out by standard addition method were found to be 100.21±1.06 and 100.04±0.30 by height and area respectively. The method was validated with the determination of accuracy, precision, specificity, linearity detector response and ruggedness. The proposed method provides a faster and cost effective quality control tool for routine analysis of content uniformity test for rosiglitazone in tablet formulation.

Rosiglitazone (RGT) is thiazolidinedione derivative is used as an antdiabetic[1]. Chemically it is 1-(N-methyl-N-pyridinyl)-2-p(5-thiazolidinedionylmethyl)phenoxyethaneamine and it is not official in any of the Pharmacopoeia. Literature survey reveals that LC[2] and automated HPLC[3] methods are reported for estimation of RGT in human plasma. Spectrophometric[4] and validated LC-UV[5] methods are reported for simultaneous estimation of rosiglitazone with glimepiride where as spectrophometric[6], RP-HPLC[7,8] and one HPTLC[9] methods are reported for estimation of rosiglitazone and gliclazide in tablet dosage form. None of these methods are content uniformity indicating. The objective of the present work was to develop and validate a sensitive and reproducible HPTLC determination and content uniformity test for the determination of low level of RGT in tablets (4 mg strength) using caffeine (CAF) as the internal standard.

Gift sample of RGT was obtained from Aristo India Ltd. Bhopal, India. All chemicals and reagents were of AR/HPLC grade. A Camag–HPTLC system comprising of Camag Linomat IV automatic sample applicator, Camag TLC Scanner III with CATS 4 software, Camag-UV cabinet and Camag twin trough glass chamber with stainless steel lids was employed. The source of radiation utilized was deuterium lamp emitting a continuous UV spectrum between 190 and 400 nm. Camag CATS 4 software offers content uniformity test method and specification according to USP. A stock solution of CAF containing 0.5 mg/ml was prepared in methanol. A mixed standard solution of RGT (1.0 mg/ml) and CAF (0.5 mg/ml) were prepared in methanol.

Experimental conditions were optimized and these were silica gel 60 GF_254_ TLC precoated aluminium foiled plates with thickness of 200 µm (E-Merck, Germany) as the stationary phase, a mixture of methanol:toluene:chloroform:triethylamine (1:8:0.5:0.5 v/v/v/v) as the mobile phase, chamber saturation time of 10 min, sample application at a constant rate of 0.16 µl/s and scanning speed 10 mm/s with 6 mm band. Ascending separation technique was used. Temperature was kept at 20±5°, relative humidity at 50-60%, migration distance at ~70 mm and the scanning mode chosen was absorbance/reflectance. Slit dimension of 5×0.45 mm was used with the detection wavelength was set at 264 nm. The detection wavelength was selected from *in situ* overlain spectra of the drugs.

For preparing a calibration curve of RGT, different volumes 1, 2, 3, 4, 5, 6 and 7 µl of standard solution of RGT (1.0 mg/ml) were applied on TLC plated using a microliter syringe with the help of an automatic sample applicator. The plates were developed, dried and densitometrically scanned at 264 nm. The curve obtained showed good linearity in the concentration range 1.0-7.0 µg/µl.

Different laboratory mixtures were prepared in the same manner as that of a mixed standard solution to get the final concentration of about 1.0 mg/ml of RGT and about 0.5 mg/ml CAF. Samples of 4.0 µl each of mixed standard solution (in duplicate) and laboratory mixture (in quadruplet) were applied to the TLC plate. Plates were then developed in presaturated twin trough chamber with mobile phase. After development the plates were dried and evaluated densitometrically.

For the assay, twenty tablets were weighed and finely powdered. The powder equivalent to 10 mg of RGT was transferred to 10.0 ml volumetric flask and shaken for 10 min with 5.0 ml methanol and volume was made to 10.0 ml. The solution was then filtered through Whatman No. 1 filter paper and filtrate was used as sample solution for assay analysis using same procedure as under laboratory mixture analysis.

The proposed method was validated according to ICH guidelines. The accuracy of proposed method was ascertained by carrying out recovery studies by standard addition method. Accurately known amount of standard drug was added to known amount of preanalysed tablet powder and it was analysed by proposed method to ascertain, if there are positive or negative interferences from excipients present in formulation. Replicate estimations of drugs in sample were carried out by proposed method and SD/RSD value was calculated as a measure of precision.

Stability indicating capability of the proposed method was investigated by applying sample to different stress condition to access the presence of components that may expect to be present, such as impurities, degradation product and matrix components. The sample solution was allowed to be stored for 24 h under the different stress conditions like 1.0 ml of 0.1 N of HCl (acid), 1.0 ml of 0.1 N of NaOH (alkali), 3% of H_2_O_2_ (oxidation), at 60° (heat), in UV-Cabinet at 265 nm (UV). After 24 h the content of flask were shaken with methanol for 10 min and the volume was made up to 10.0 ml, filtered, diluted and analyzed following the assay procedure. Ruggedness was carried out under the different conditions i.e., analyzing the sample on different days and by different analysts.

The content uniformity test was carried out by taking 10 individual tablets of marketed formulation and was determined after extracting with methanol, which complies with the USP specification of the content uniformity test of 85-115% and % CV of 6.0.

The mobile phase, methanol:toluene:chloroform:triethylamine (1:8:0.5:0.5 v/v/v/v) yielded good resolution of the analytes under investigation on silica gel 60 GF_254_ TLC plate with R_f_ values of 0.31 for RGT and 0.52 for CAF at 264 nm (fig. 1). The other parameters as detailed under chromatographic condition were optimized on the basis of exhaustive experimentation. Plots of concentration Vs peak height/peak area have been linear over concentration range 1-7 µg with coefficient of correlation 0.9888. The results of replicate estimation of drugs in tablet were quite concurrent indicating the precision. The recovery of the drugs form the sample matrix evaluated on the basis of standard addition have been almost about 100 % indicating the accuracy of the method and non interference of the sample matrix (Table 1). The results of estimation of samples subjected to various stress conditions were quite comparable to normal samples. This is indicative that there is no degradation of the sample under stress conditions and excipients present in the formulation did not interfere. The estimation of samples on different days and by different analysts shows reproducibility of results indicating ruggedness of the method. The summary of validation parameter is listed in Table 2. The diagrammatic representation generated using Camag CATS 4 software single level content uniformity option for 10 units of tablet formulation for RGT by area are shown in fig. 2. Since the content of individual tablet unit and % CV between the content of 10 tablet units fall within the permissible limits according to USP, tablet formulation comply with the content uniformity test of USP.

**Fig. 1:**
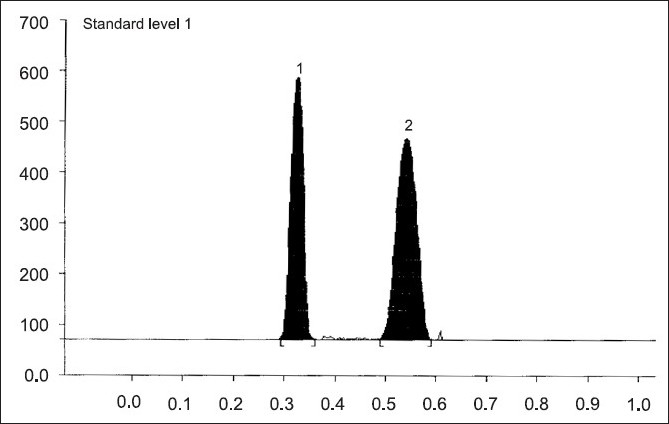
Densitogram of rosiglitazone and internal standard caffeine at 264 nm

**Fig. 2:**
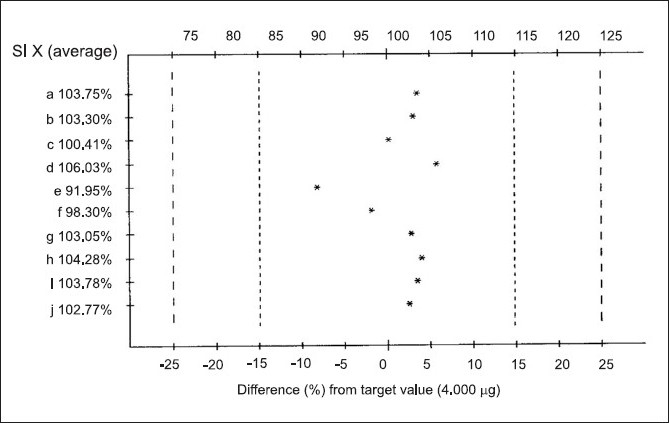
Percentage content in ten individual tablets of marketed formulation Diagrammatic representation of percentage content in ten individual tablet of units of marketed formulation (linear regression as per peak area rosiglitazone, USP limits for drug content is 85 -115%)

**TABLE 1:** ESTIMATION OF RGT IN LABORATORY MIXTURE, MARKETED FORMULATION AND RECOVERY STUDY

Sample	Statistics	% Estimation*		% Recovery*	
		RGT		RGT	
		By height	By area	By height	By area
Standard Laboratory Mixture	Mean	100.12	99.71	-------	-------
	±SD	0.6605	0.7138	-------	-------
	CV	0.6597	0.7159	-------	-------
Marketed Formulation1	Mean	100.02	100.21	-------	-------
	±SD	0.6774	0.2643	-------	-------
	CV	0.6773	0.2637	-------	-------
Marketed Formulation2	Mean	100.02	99.83	100.21	100.04
	±SD	0.5388	0.2722	1.0665	0.3070
	CV	0.5387	0.2727	1.0643	0.3069

Summary of estimation of RGT in laboratory mixture, marketed formulation and recovery study

* Each reading is the mean of five observations, RGT is rosiglitazone, SD is standard deviation and CV is coefficient of variance

**TABLE 2:** VALIDATION PARAMETERS

Parameter	Value	
Linearity range	1.0‐7.0 µg/µl	
Coefficient of correlation	0.9888±0.002	
Specificity	Specific	
Precision (% C.V.)	By height	By area
Different days (n=3)	0.3247	0.4972
Different analysts (n=3)	0.9809	0.3942

In general the method is simple, accurate, precise, specific and rugged and may be adopted for routine estimation of RGT in single dose formulation. As proposed method can analyze 10 tables on single plate simultaneously, it proves to be very fast and cost effective and can be employed for determination of content uniformity of RGT in tablet dosage form on routine basis.
